# Genome Sequence of *Streptococcus phocae* subsp. *phocae* Strain ATCC 51973^T^ Isolated from a Harbor Seal (*Phoca vitulina*)

**DOI:** 10.1128/genomeA.01307-15

**Published:** 2015-11-19

**Authors:** Ruben Avendaño-Herrera, Matías Poblete-Morales

**Affiliations:** a Facultad de Ciencias Biológicas, Departamento de Ciencias Biológicas, Laboratorio de Patología de Organismos Acuáticos y Biotecnología Acuícola, Universidad Andrés Bello, Viña del Mar, Chile; b Interdisciplinary Center for Aquaculture Research (INCAR), Concepción, Chile; c Centro de Investigación Marina Quintay, CIMARQ, Quintay, Chile

## Abstract

*Streptococcus phocae* subsp. *phocae* is a pathogen that affects different pinniped and mammalian species. This announcement reports the genome sequence of the type strain ATCC 51973 isolated in Norway from clinical specimens of harbor seal (*Phoca vitulina*), revealing interesting genes related to possible virulence factors.

## GENOME ANNOUNCEMENT

*Streptococcus phocae* is a beta-hemolytic Gram-positive bacterium and member of the pyogenic streptococcal group (1). It was first isolated in Norway from clinical specimens of harbor seal (*Phoca vitulina*) (2). This pathogen has since been identified in different seal species from several countries (3–6), and in 2005, this bacterium was isolated during disease outbreaks at an Atlantic salmon (*Salmo salar*) farm (7).

A recent comparative polyphasic study analyzed strains from different host origins to clarify the taxonomic position of strains isolated from Atlantic salmon. This study reclassified *S. phocae* as *S. phocae* subsp. *salmonis*, thus becoming the only subspecies apart from *S. phocae* subsp. *phocae* to be isolated from seals. These two subspecies differ biochemically and physiologically, including in hemolytic capacities, range of growth temperature, and host specificity (8). The type strain of *S. phocae* subsp. *phocae* is ATCC 51973 (2).

The *S. phocae* subsp. *phocae* ATCC 51973^T^ genome was sequenced from a DNA library using the Illumina Nextera XT library preparation protocol, according to the manufacturer’s instructions, and MiSeq technology with 50-fold coverage. The MiSeq reads were *de novo* assembled into 111 contigs using the CLC Genomics Workbench version 8.0.2, with 92 contigs >200 bp. The estimated length of the chromosome was 1,700,445 bp, with a G+C content of 39.5%, which is very close to that described by Skaar et al. (2), who reported a G+C content of 38.6% and a total of 1,754 predicted open reading frame regions.

Whole-genome analysis of ATCC 51973^T^ revealed gene sets related to information subsystems coding for binding factors, such as the fibronectin-binding protein, and toxins, such as streptolysin S, which is a recognized virulence factor of *Streptococcus pyogenes* (9). Capsule and extracellular polysaccharides were also found, which would allow the bacteria to damage host tissues (10). Moreover, the genome sequence included genes resistant to fluoroquinolone, such as topoisomerase IV subunits A and B, DNA gyrase subunits A and B, and the multiantimicrobial extrusion protein. Importantly, hyaluronidase was also found, which codes for integrated phages in the host chromosome. In other *Streptococcus* species, hyaluronidase influences pathogenicity and is directly associated with bacteria propagation during infection (11, 12). This genome sequence will facilitate comprehensive bioinformatics and phylogenetic analyses, thus expanding understandings on the pathogenesis of *S. phocae* subsp. *phocae*.

### Nucleotide sequence accession number.

The complete genome sequence has been deposited in DDBJ/EMBL/GenBank under the accession no. LHQM00000000. The version described in this paper is the second version.
